# Novel small molecule SIRT2 inhibitors induce cell death in leukemic cell lines

**DOI:** 10.1186/s12885-018-4710-1

**Published:** 2018-08-06

**Authors:** Tomohiro Kozako, Paolo Mellini, Takeo Ohsugi, Akiyoshi Aikawa, Yu-ichiro Uchida, Shin-ichiro Honda, Takayoshi Suzuki

**Affiliations:** 1 Department of Biochemistry, Faculty of Pharmaceutical Sciences, Fukuoka University, 8-19-1 Nanakuma, Jonan-ku, Fukuoka, 814-0180 Japan; 2 Faculty of Medicine, Kyoto Prefectural University of Medicine, Kyoto, Japan; 3 Department of Hematology and Immunology, Rakuno Gakuen University, Hokkaido, Japan; 4 CREST, Japan Science and Technology Agency (JST), Saitama, Japan

**Keywords:** Human T-cell leukemia virus-1, Adult T-cell leukemia/lymphoma, SIRT2, Apoptosis, Caspase-independent cell death

## Abstract

**Background:**

Sirtuin 2 (SIRT2) is a member of the sirtuin family, nicotinamide adenine dinucleotide^+^-dependent deacylases, which participates in modulation of cell cycle control, neurodegeneration, and tumorigenesis. SIRT2 expression increases in acute myeloid leukemia blasts. Downregulation of SIRT2 using siRNA causes apoptosis of HeLa cells. Therefore, selective inhibitors of SIRT2 are candidate therapeutic agents for cancer. Adult T-cell leukemia/lymphoma (ATL) is a T-cell malignancy that has a poor prognosis and develops after long-term infection with human T-cell leukemia virus (HTLV)-1. Sirtuin 1 inhibition has been shown to induce apoptosis and autophagy in HTLV-1-infected cell lines, whereas the effects of SIRT2 inhibition alone have not been elucidated.

**Methods:**

We assessed the efficacy of our small molecule selective SIRT2 inhibitors NCO-90/141 to induce leukemic cell death. Cell viability was examined using the cell proliferation reagent Cell Count Reagent SF. Apoptotic cells were detected by annexin V-FITC and terminal deoxynucleotidyl transferase dUTP nick end labeling assays by flow cytometry. Caspase activity was detected using an APOPCYTO Intracellular Caspase Activity Detection Kit. The presence of autophagic vacuoles was assessed using a Cyto-ID Autophagy Detection Kit.

**Results:**

Our novel small molecule SIRT2-specific inhibitors NCO-90/141 inhibited cell growth of leukemic cell lines including HTLV-1-transformed T-cells. NCO-90/141 induced apoptosis via caspase activation and mitochondrial superoxide generation in leukemic cell lines. However, a caspase inhibitor did not prevent this caspase-associated cell death. Interestingly, NCO-90/141 increased the LC3-II level together with autophagosome accumulation, indicating autophagic cell death. Thus, NCO-90/141 simultaneously caused apoptosis and autophagy.

**Conclusions:**

These results suggest that NCO-90/141 are highly effective against leukemic cells in caspase-dependent or -independent manners via autophagy, and they may have a novel therapeutic potential for treatment of leukemias including ATL.

**Electronic supplementary material:**

The online version of this article (10.1186/s12885-018-4710-1) contains supplementary material, which is available to authorized users.

## Background

Sirtuins (SIRT1–7) are nicotinamide adenine dinucleotide^+^-dependent deacylases or mono-[ADP-ribosyl] transferases that display diverse subcellular localizations and functions [1–3]. SIRT2 has an essential role in maintaining the integrity of mitosis and has been proposed to act as a tumor suppressor by preventing chromosomal instability during mitosis [4]. However, tumors that express high levels of SIRT2 are resistant to chemotherapy, specifically microtubule toxins [5]. SIRT2 mRNA levels are significantly elevated in acute myeloid leukemia (AML) blasts compared with those in bone marrow from healthy individuals [6]. High expression of SIRT2 is also an unfavorable prognostic biomarker for AML risk stratification [7]. A recent study has shown that pharmacological inhibition of both SIRT1 and SIRT2 reduces cell viability by apoptosis in adult T-cell leukemia/lymphoma (ATL) cells and delays tumor growth through p53 activation in melanoma [8, 9].

ATL is a T-cell malignancy derived from mature CD4^+^ T-cells and has a poor prognosis, which develops after long-term infection with human T-cell leukemia virus (HTLV)-1 [10–12]. Although the underlying mechanisms of ATL development have not been fully elucidated, genetic and epigenetic abnormalities have been implicated [13–16]. There are four subtypes of ATL, including acute, lymphoma, chronic, and smoldering [17]. Despite recent advances in chemotherapy, allogeneic hematopoietic stem cell transplantation, and antibody therapy, the prognoses of patients with acute lymphoma types are still unsatisfactory [18–21]. Therefore, there is a clear need for new molecular targets for the development of treatments for ATL.

We previously reported that NCO-01 and NCO-04 inhibit both SIRT1 and SIRT2 activities in enzyme assays and induce apoptotic cell death [8, 22]. SIRT1 and SIRT2 inhibition has been shown to induce apoptosis and autophagy, whereas the effects of SIRT2 inhibition alone have not been elucidated. In this study, we assessed the efficacy of our small molecule selective SIRT2 inhibitors NCO-90/141 to induce leukemic cell death. We found that NCO-90/141 induced apoptotic cell death by caspase activation in leukemic cell lines and induced caspase-independent cell death (CICD) by autophagosome accumulation and autophagy. This is the first evidence demonstrating the cell growth-inhibiting effect of SIRT2-specific inhibitors via caspase-dependent or -independent cell death such as autophagy in leukemic cells.

## Methods

### Cell lines

Cell lines S1T (HTLV-1-infected CD4^+^ T-cell line derived from an ATL patient; kindly provided by Dr. Naomichi Arima, Kagoshima University), [23] MT-2 (HTLV-1-infected T-cell line derived from normal human leukocytes transformed by leukemic T-cells from an ATL patient) purchased from Japanese Cancer Research Resources Bank (Osaka, Japan; catalogue number: JCRB1210), [24] Jurkat (T-lineage acute lymphoblastic leukemia cell line) purchased from RIKEN BioResource center (BRC) (Ibaraki, Japan; catalogue number: RBRC-RCB3053), and HL60 (acute myeloid leukemia cell line) purchased from RIKEN BRC (catalogue number: RBRC-RCB0041) were cultured in RPMI-1640 medium supplemented with 10% heat-inactivated fetal calf serum, 2 mM l-glutamine, 0.1 mg/mL streptomycin, and 100 U/mL penicillin [8].

### Reagents

**Fig. 1:**
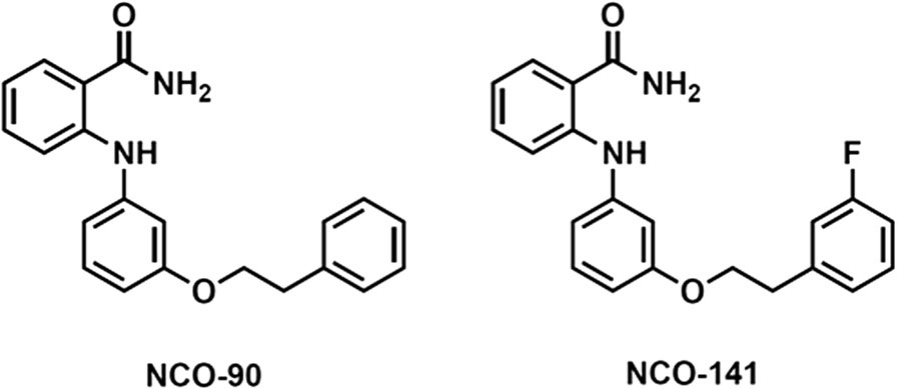
Chemical structures of NCO-90/141

The novel SIRT2 inhibitors evaluated in this study, NCO-90 and NCO-141, have been described in a previous report [25]. Chemical structures of NCO-90 and NCO-141 are shown in Fig. 1. Molecular weights of NCO-90 and NCO-141 are 332.40 and 350.39, respectively. Caspase inhibitor Z-VAD-FMK and anti-Fas monoclonal antibody CH11 were purchased from Medical and Biological Laboratories (MBL) (Nagoya, Japan). STF-62247, an autophagy inducer, was purchased from Calbiochem (Darmstadt, Germany). Bafilomycin A1, a specific inhibitor of vacuolar proton ATPase, was purchased from Adipogen (Épalinges, Switzerland).

We purchased primary antibodies against β-actin, histone H3, p53, acetyl-p53K382 (ac-p53), and SIRT2 from Cell Signaling Technology (Beverly, CA), a primary antibody against acetyl-histone H4K16 (ac-H4) from Abcam (Cambridge, UK), and a primary antibody against LC3 from MBL. Horseradish peroxidase-conjugated secondary antibodies were purchased from Vector Laboratories (Burlingame, CA).

### Protein extraction and western blot analysis

Cell lysates were obtained using RIPA Lysis Buffer RIPA Lysis Buffer (Santa Cruz Biotechnology, Dallas, TX). Nuclear extracts were obtained using NE-PER Nuclear and Cytoplasmic Extraction Reagents (Pierce Biotechnology, Rockford, IL), according to the manufacturer’s protocol. Western blotting was performed as described previously [26]. Images were obtained and analyzed using a ChemiDoc™ XRS (Bio-Rad, Hercules, CA).

### Cell viability assay

Cell viability was examined using the cell proliferation reagent Cell Count Reagent SF (Nacalai Tesque) as described previously [8]. The half maximal inhibitory concentration for cell growth (GI_50_) was calculated using Grafit (Erithacus Software, Horley, UK).

### Apoptosis analysis

Apoptotic cells were stained with annexin V-FITC (MBL) and 7-amino-actinomycin D (Beckman Coulter, Brea, CA), and analyzed by flow cytometry using a Cell Analyzer EC800 (Sony, Tokyo, Japan) as described previously [27, 28]. The percentages of specific apoptotic cells were calculated as follows: % specific apoptotic cells = (annexin V-positive cells − spontaneous annexin V-positive cells)/(100 − spontaneous annexin V-positive cells) × 100.

DNA fragmentation was detected by terminal deoxynucleotidyl transferase dUTP nick end labeling (TUNEL) assays using a MEBSTAIN Apoptosis Kit Direct (MBL) [8].

### Mitochondrial superoxide generation assay

Measurements of mitochondrial superoxide generation were performed using MitoSOX Red (Molecular Probes, Invitrogen) as described previously [8].

### Detection of caspase activity

Caspase activity was detected using an APOPCYTO Intracellular Caspase-3 or − 8 Activity Detection Kit (MBL) and CaspGLOW Fluorescein Active Caspase-9 Staining Kit (BioVision, Milpitas, CA) as described previously [8].

### Analysis of autophagy by flow cytometry

The presence of autophagic vacuoles was assessed using a Cyto-ID Autophagy Detection Kit (Enzo Life Sciences, Farmingdale, NY), according to the manufacturer’s instructions [29, 30]. Autophagy analysis was performed by incubating cells with bafilomycin A1 for 30 min at 37 °C prior to treatment with CYTO-ID Green Detection Reagent, and then analyzing fluorescence by flow cytometry using the Cell Analyzer EC800 as described previously [8]. Autophagy was also evaluated using the FlowCellect™ Autophagy LC-3 Antibody-based Assay kit (Merck Millipore) to monitor lapidated LC-3-II as described previously [8]. Quantification of anti-LC3-FITC fluorescence was performed by pretreatment with a lysosomal inhibitor for 30 min prior to treatment of the 48-h NCO-90/141-treated sample to prevent lysosomal degradation of LC3.

### Statistical analysis

Data are expressed as means ± SD. For data analyses, two-tailed Student’s *t*-tests and Wilcoxon matched-pairs tests were performed using Excel 2010 (Microsoft Japan, Tokyo, Japan) and Statcel2 software (OMS Publishing Inc., Tokyo, Japan). In all tests, values of *P* < 0.05 were considered as statistically significant.

## Results

### NCO-90/141 inhibit the cell growth of leukemic cell lines

**Fig. 2:**
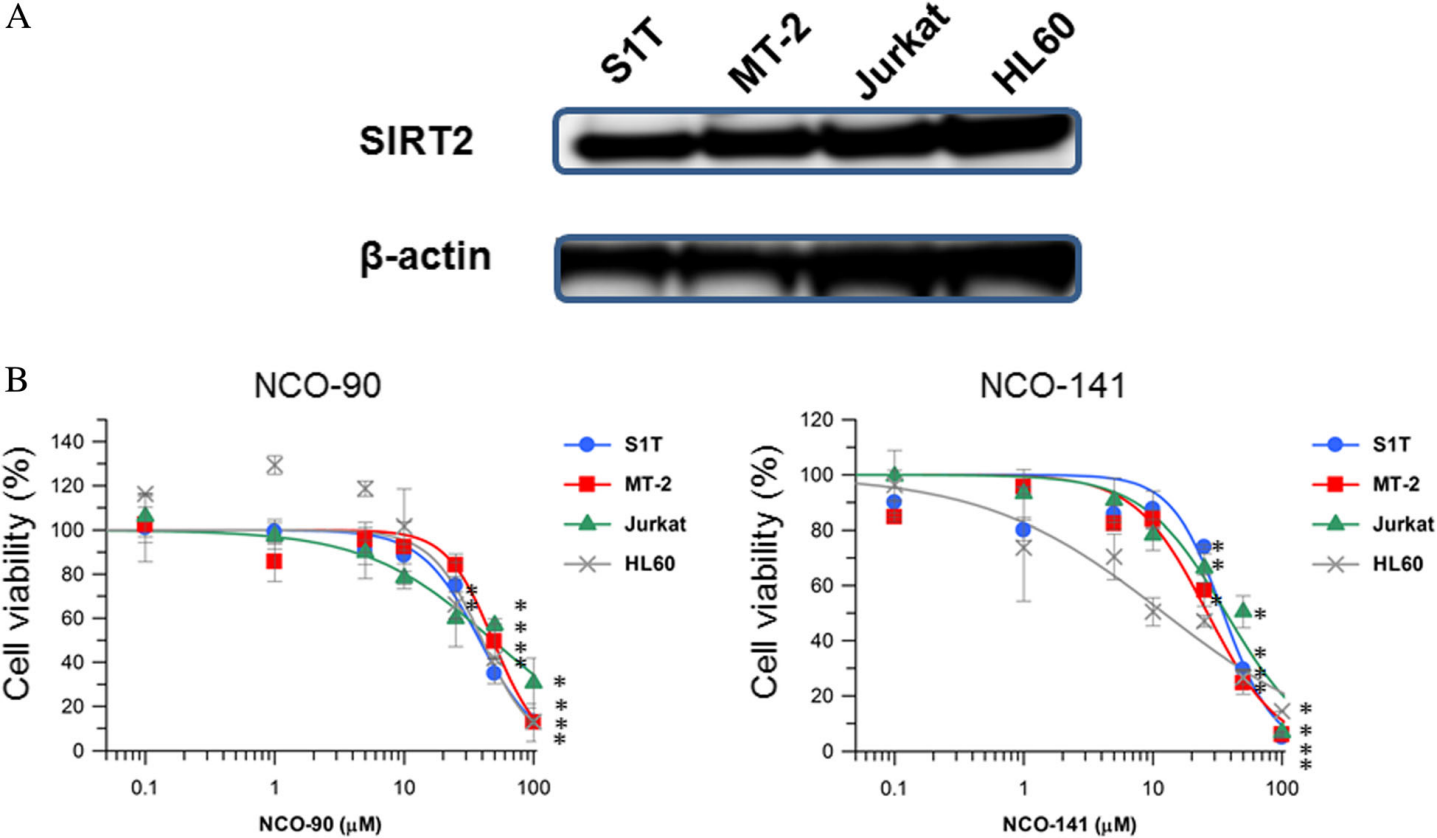
NCO-90/141 reduce cell viability in leukemic cell lines. **a** SIRT2 protein in leukemic cell lines was analyzed by western blotting. **b** Cell lines were incubated at 2 × 10^5^ cells/mL in the presence of various concentrations of NCO-90 and NCO-141 for 72 h. The viabilities of the cultured cells were measured by Cell Count Reagent SF. Cells cultured in the absence of each SIRT2 inhibitor were assigned a relative viability of 1. Data represent mean percentages ± SD of three independent experiments. *P < 0.05 vs. 0 μM

We examined whether NCO-90/141 affected the cell growth of S1T, MT-2, Jurkat, and HL60 cells that express SIRT2 protein (Fig. 2). Our small molecule selective SIRT2 inhibitors showed potent activities with IC_50_ values of 1.0 and 0.5 μM for NCO-90 and NCO-141, respectively, whereas NCO-90/141 did not inhibit SIRT1 at up to 300 μM. NCO-90/141 inhibited the growth of all four cell lines in a dose-dependent manner. NCO-90 showed potent activities with GI_50_ values of 38.3, 48.5, 48.2, and 40.2 μM in S1T, MT-2, Jurkat, and HL60 cells, respectively. NCO-141 showed potent activities with GI_50_ values of 34.9, 26.4, 36.7, and 12.1 μM in S1T, MT-2, Jurkat, and HL60 cells, respectively. In contrast, commercially available SIRT2 inhibitor AKG2 had no GI_50_ values in leukemic cell lines (Additional file 1: Figure S1). AKG2 inhibited SIRT1 and SIRT2 with IC_50_ values of 30 and 3.5 μM, respectively.

### NCO-90/141 induce apoptosis by caspase activation and mitochondrial superoxide generation in leukemic cell lines

**Fig. 3:**
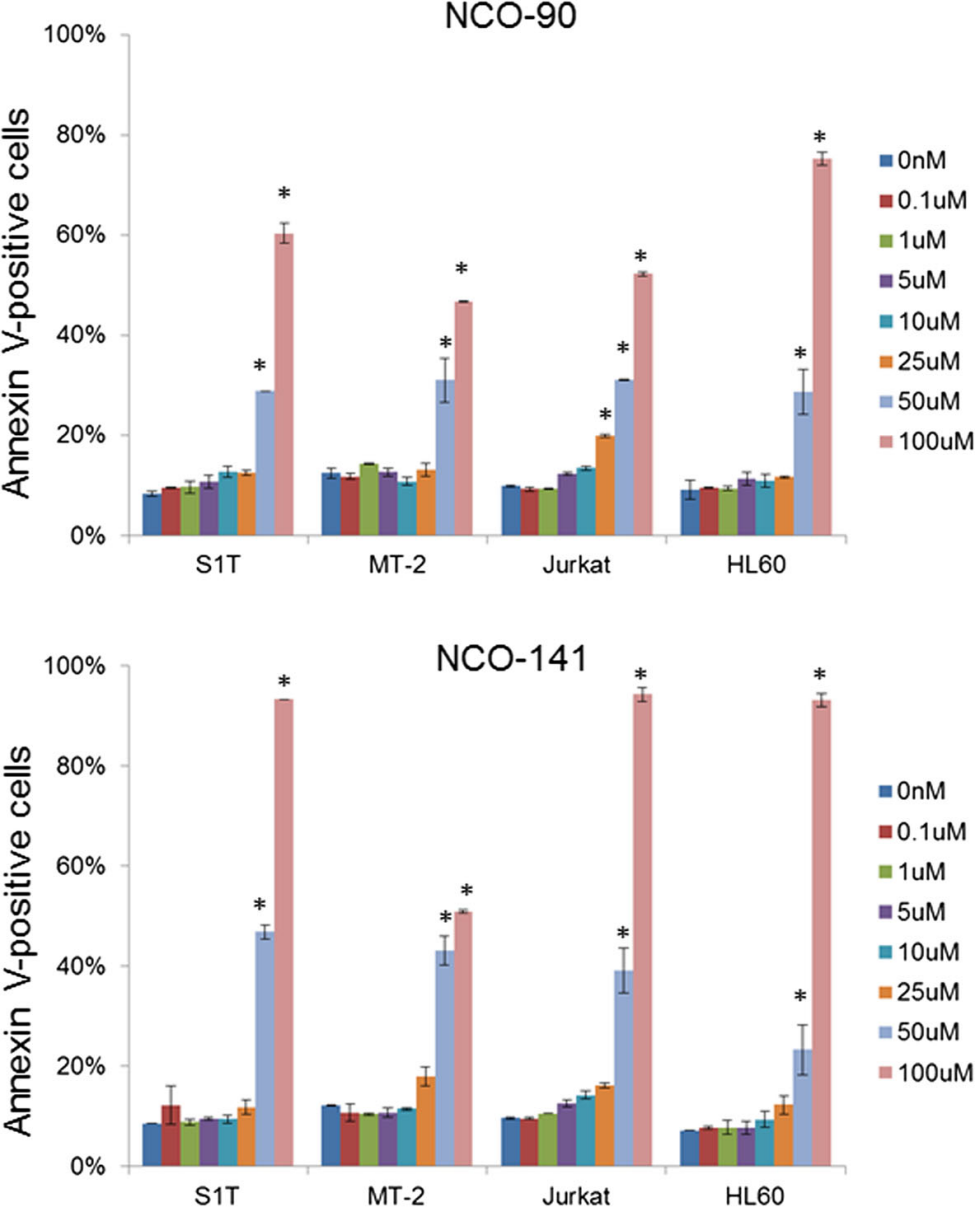
NCO-90/141 induce annexin V-positive cells in leukemic cell lines. Cell lines were incubated at 2 × 10^5^ cells/mL in the presence of various concentrations of NCO-90 and NCO-141 for 72 h. Annexin V-positive cells were detected by flow cytometry. Data represent mean percentages ± SD of apoptotic cells in three independent experiments. *P < 0.05 vs. 0 μM

To examine whether NCO-90/141 induce apoptosis, we analyzed NCO-90/141-induced cell death by annexin V staining (Fig. 3). We observed that NCO-90/141 induced annexin V-positive cells in leukemic cell lines. The percentages of specific annexin V-positive cells induced by NCO-90 (100 μM) were 52.5%, 34.7%, 42.3%, and 66.5% in S1T, MT-2, Jurkat, and HL60 cell lines, respectively. The percentages of specific annexin V-positive cells induced by NCO-141 (100 μM) were 85.3%, 39.0%, 85.9%, and 86.7% in S1T, MT-2, Jurkat, and HL60 cell lines, respectively. NCO-90/141 induced the early phase of apoptosis (annexin V^+^/7-amino-actinomycin D^−^; Fig. 5b). We also observed that NCO-90/141 induced DNA fragmentation and caspase activation in the leukemic cell lines (Figs. 5c and 6).

**Fig. 4:**
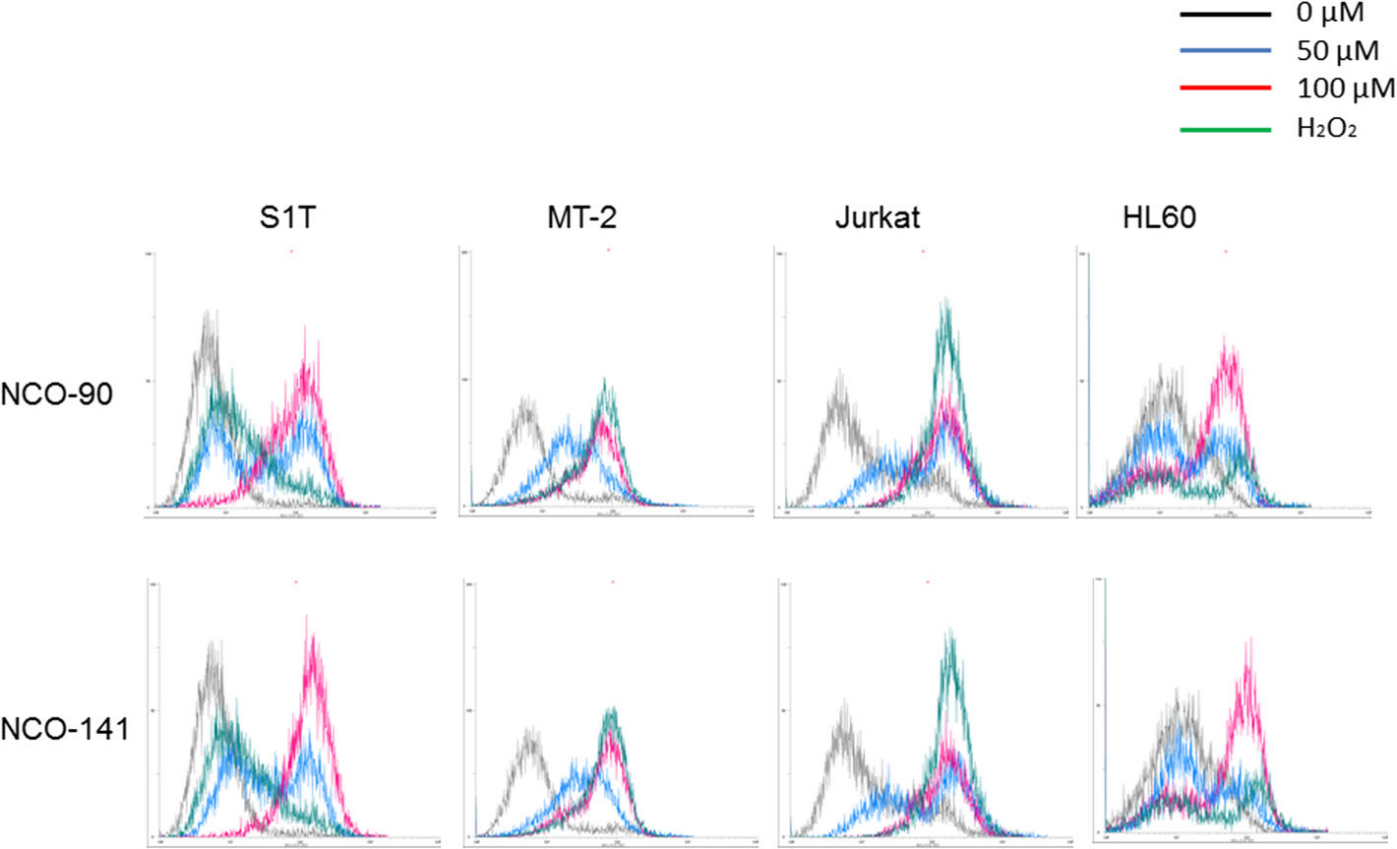
NCO-90/141 induce mitochondrial superoxide generation in leukemic cell lines. S1T, MT-2, Jurkat, and HL60 cells were treated with NCO-90 or NCO-141 for 6 h and then analyzed using MitoSOX Red by flow cytometry

During apoptosis, mitochondria play key roles including the release of caspase activators such as cytochrome c following loss of mitochondrial transmembrane potential [31]. In addition to this essential role of mitochondria during the execution phase of apoptosis, it appears that reactive oxygen species produced by mitochondria are involved in cell death [32]. MitoSOX Red detects superoxides in mitochondria of live cells. The positive charge on the phosphonium group in MitoSOX Red selectively targets this cell-permeable hydroethidine derivative to mitochondria where it accumulates as a function of mitochondrial membrane potential and exhibits fluorescence upon oxidation and subsequent binding to mitochondrial DNA. By measuring the shift in fluorescence emission by flow cytometry, mitochondrial superoxide generation was detected in NCO-90/141-treated cells (Fig. 4).

### NCO-90/141 induce both caspase-dependent and -independent cell death

**Fig. 5:**
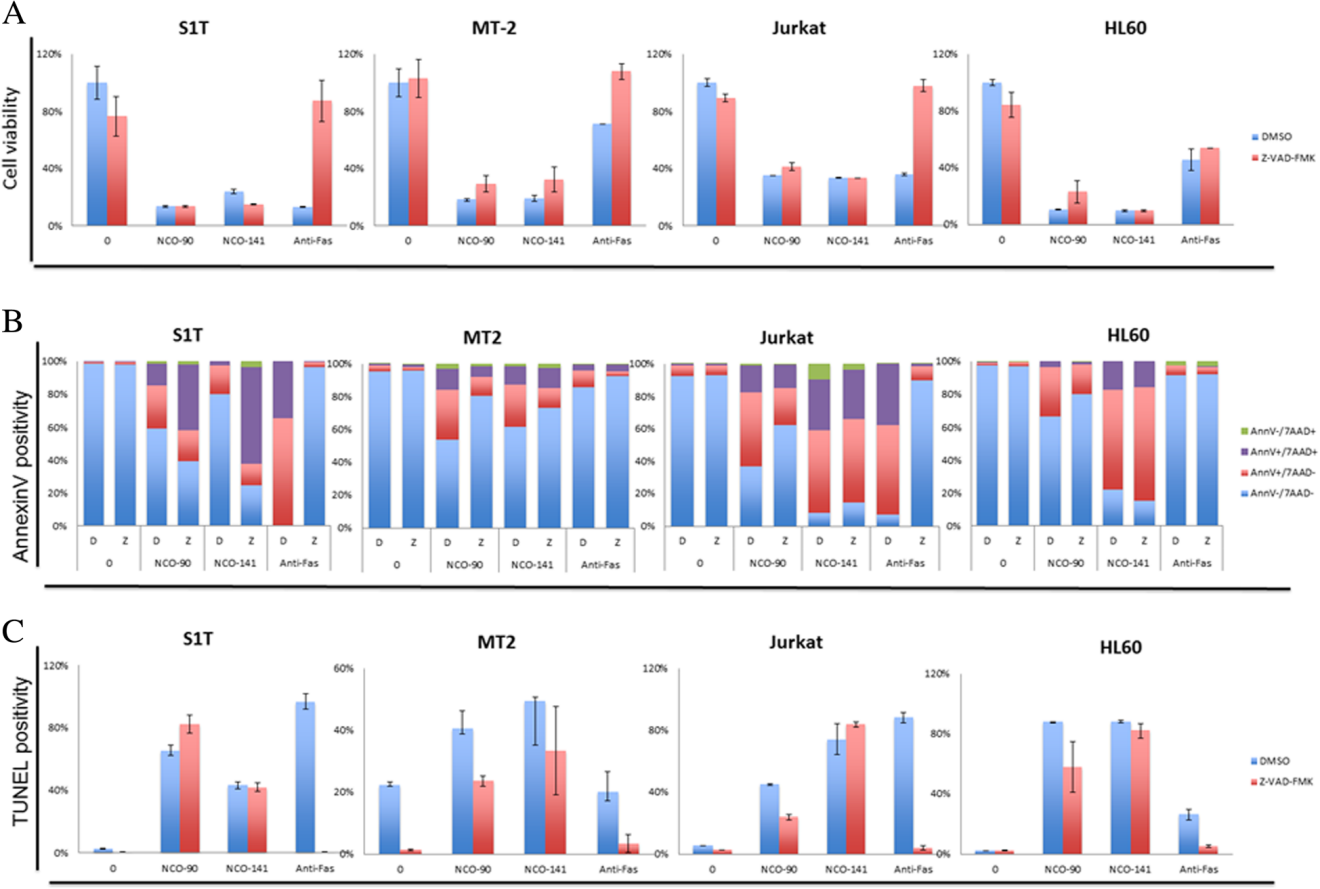
NCO-90/141 induce caspase-independent cell death. S1T, MT-2, Jurkat, and HL60 cells were treated with NCO-90 (50 μM) or NCO-141 (S1T and MT-2 cells, 50 μM; Jurkat and HL-60 cells, 100 μM) and Z-VAD-FMK (40 μM) for 72 h. **a** Viability of cultured cells was measured by Cell Count Reagent SF. **b** and **c** Annexin V-, 7-amino-actinomycin D (7AAD)-, and TUNEL-positive cells were detected by flow cytometry. Data represent mean percentages ± SD of three independent experiments

**Fig. 6:**
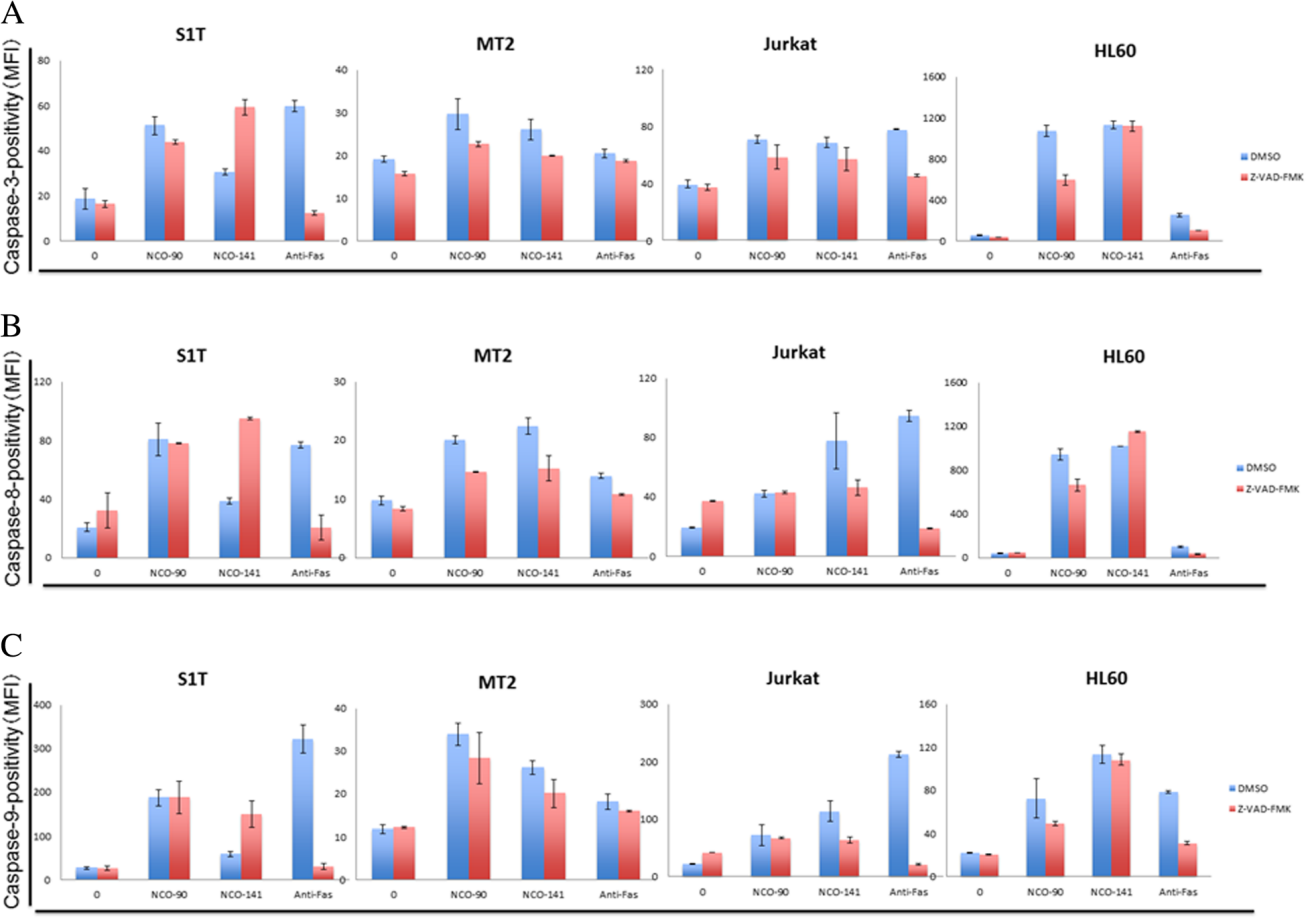
NCO-90/141 induce both caspase-dependent and -independent cell death with caspase activities. S1T, MT-2, Jurkat, and HL60 cells were treated with NCO-90 (50 μM) or NCO-141 (S1T and MT-2 cells, 50 μM; Jurkat and HL-60 cells, 100 μM) and Z-VAD-FMK (40 μM) for 72 h. **a**–**c** Caspase-positive cells were detected by flow cytometry. Data represent mean percentages ± SD of three independent experiments

These data suggested that NCO-90/141 might induce apoptosis via a caspase-dependent pathway. To test this, we assessed the effects of a pan-caspase inhibitor, Z-VAD-FMK, on NCO-90/141-induced cell death (Figs. 5 and 6). NCO-90/141 induced significant growth inhibition with annexin V-positive cells and DNA fragmentation in leukemic cell lines. NCO-90/141 also activated caspase activity (caspase-3, − 8, and − 9). Z-VAD-FMK was able to suppress anti-Fas antibody-induced cell death as a positive control. However, Z-VAD-FMK, which inhibits caspase-1, − 3, − 4, − 7, and − 8, did not suppress the cell death, annexin V-positive cells, or DNA fragmentation (Fig. 6a–c). Z-VAD-FMK also did not inhibit caspase-3, − 8, or − 9 activities (Fig. 6a–c).

**Fig. 7:**
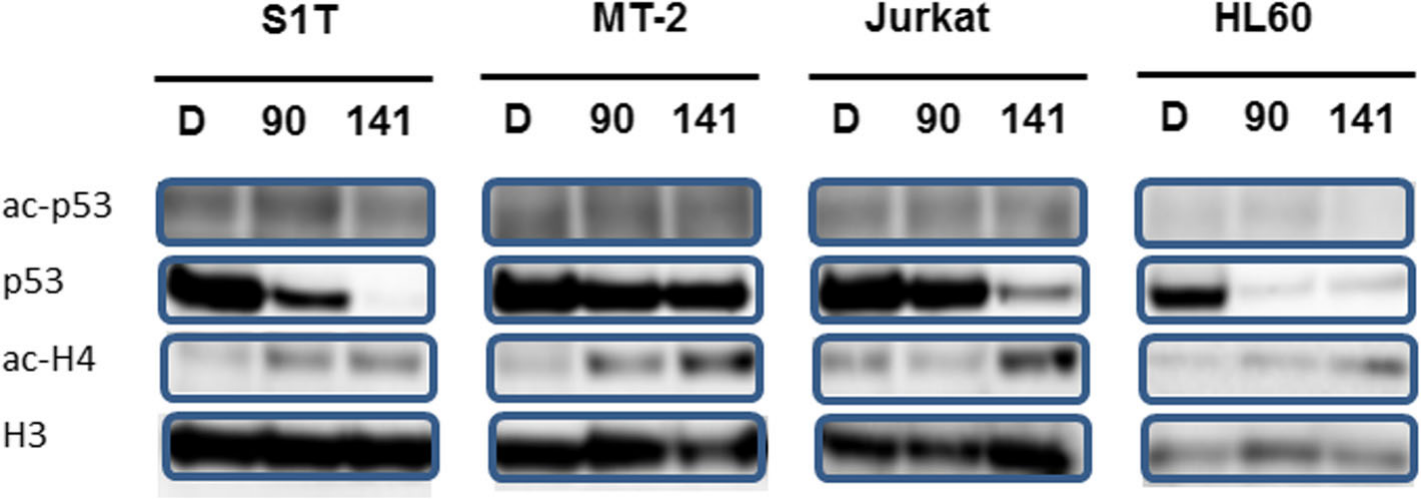
NCO-90/141 induce p53 degradation. S1T, MT-2 Jurkat, and HL60 cells were treated with NCO-90 or NCO-141 for 72 h. Protein levels in the nucleus were detected by western blotting with the indicated antibodies

An early increase in reactive oxygen species has been found to precede mitochondrial membrane permeabilization, and in some case to be independent of caspases in various models including apoptosis induced by p53 [32]. Acetylation of p53 induced by cellular stress stimulates the DNA-binding capacity of p53 and enhances its biological functions. NCO-90/141 increased levels of acetylated H4, which is a SIRT2 substrate, but did not increase acetylated p53 (Fig. 7). In contrast, degradation of p53 in the nucleus was increased by NCO-90/141.

### NCO-90/141 induce autophagy in leukemic cell lines

**Fig. 8:**
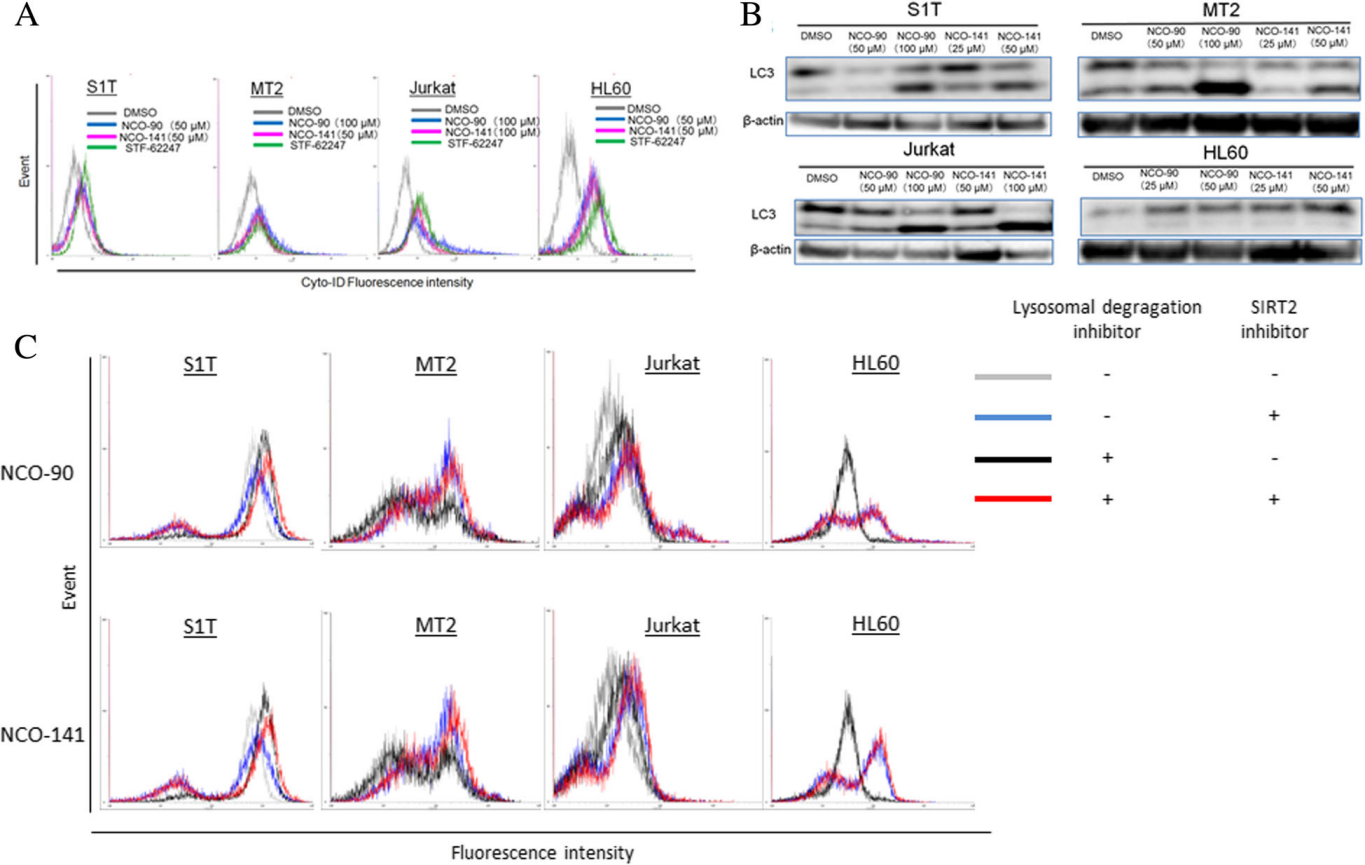
NCO-90/141 induce autophagy. S1T, MT-2, and Jurkat cells were treated with NCO-90/141 and STF-62247 (50 μM), an autophagy inducer, for 48 h. **a** Autophagy was measured by quantifying the mean fluorescence intensity using a Cyto-ID Autophagy Detection Kit. **b** Protein levels were detected by western blotting with the indicated antibodies. **c** Cellular autophagic flux monitored by lapidated LC3-II was evaluated using the FlowCellect™ Autophagy LC3 Antibody-based Assay Kit. S1T, MT-2 Jurkat, and HL60 cells were treated with 50 μM NCO-90 (upper) or NCO-141 (lower) for 24 h

Some mitochondrial proteins, such as apoptosis-inducing factor, which are released as a result of mitochondrial outer membrane permeabilization, promote CICD [33]. The incidence of CICD concomitant with increased autophagic activity may be indicative of autophagic type II cell death [34]. Autophagy degrades cellular components, so that cells eventually activate their apoptosis machinery [35]. Autophagy was detected using a Cyto-ID Autophagy Detection Kit [29]. Autophagy levels were increased in the presence of NCO-90/141 (Fig. 8a). A shift of the soluble form LC3-I to the autophagic vesicle-associated form LC3-II is a specific marker for autophagosome promotion. NCO-90/141 significantly increased the levels of LC3-II (lapidated LC3; Fig. 8b). Thus, NCO-90/141 increased autophagosome accumulation and autophagy in leukemic cell lines. Next, we confirmed whether NCO-90/141 increased activation of autophagic flux or inhibited autophagosome degradation (Fig. 8c). The shift of the LC-3 level was observed after NCO-90/141 treatment together with the lysosome inhibitor (red) in comparison with no treatments (black). NCO-90/141 induced LC3 translocation. However, there was a shift of the LC-3 level by treatment with NCO-90/141 in the absence of the lysosome inhibitor (blue) in comparison with no NCO-90/141 treatment (gray). Thus, NCO-90/141 also inhibited autophagosome degradation.

## Discussion

SIRT2, a nicotinamide adenine dinucleotide^+^-dependent deacetylase, has been proposed to be a tumor suppressor associated with aging, the cell cycle, and carcinogenesis [36]. SIRT2 knockout mice develop cancers in multiple organs via aurora-A and -B that direct centrosome amplification, aneuploidy, and mitotic cell death [4]. However, SIRT2 mRNA levels are significantly elevated in AML blasts [6]. SIRT2-overexpressing cells also exhibit prolongation of the cell cycle [37]. SIRT2 activity in glioma cells is required for survival [38]. Furthermore, SIRT2 downregulation using siRNA causes apoptosis of HeLa cells [39]. Therefore, SIRT2 inhibitors are emerging as antitumor drugs [40]. Here, SIRT2 protein expression in leukemic cell lines indicated that SIRT2 is a target for treatment of leukemia (Fig. 2). We have developed specific SIRT2 inhibitors NCO-90 and NCO-141 [22]. NCO-90/141 inhibited cell growth of leukemic cell lines including HTLV-1-transformed T cells by apoptosis and CICD (Figs. 2, 3, 4, 5 and 6). Selective SIRT2 inhibitors induce cell death in non-small cell lung cancer and breast cancer cell lines [41, 42]. Thus, SIRT2 inhibitors are promising lead candidates for use in cancer treatments. However, studies of SIRT2 functions in cancers have obtained contradictory results, indicating that further studies will be required to estimate the therapeutic potential of targeting SIRT2 in cancer [1, 36, 43].

In this study, a caspase inhibitor did not prevent NCO-90/141-induced cell death, indicating that NCO-90/141 induced CICD (Figs. 5 and 6). Under the conditions of CICD, glyceraldehyde-3-phosphate dehydrogenase in glycolysis participates in transcriptional upregulation of ATG12 and enhances autophagy [34]. Furthermore, caspase inhibition often leads to autophagic cell death when caspase inhibition does not inhibit cell death [33]. Autophagy is the regulated and destructive mechanism by which long-lived proteins, organelles, and protein aggregates are captured within autophagosomes [44–46]. Here, we found that NCO-90/141 inhibited the growth of leukemic cell lines and increased LC3-II levels by mitochondrial superoxide generation and caspase activation (Figs. 4 and 8). Autophagy caused by NCO-90/141 may induce degradation of p53. SIRT2 interferes with autophagy-mediated degradation of protein aggregates in neuronal cells under proteasome inhibition [47]. SIRT2 knockdown also increases basal autophagy [48]. Mitochondrial outer membrane permeabilization triggers the removal of permeabilized mitochondria by the autophagic machinery [49]. Thus, SIRT2 inhibition induces autophagy by mitochondrial superoxide generation. Furthermore, NCO-90/141-induced cell death was not inhibited in combination with bafilomycin A, an autophagy flux inhibitor, for 72 h (data not shown). Therefore, these results suggest that the molecules involved in caspase-independent DNA fragmentation may augment caspase activity, and secondary caspase activation and autophagy induced by NCO-90/141 may be the result, but not the cause, of cell death.

## Conclusions

In the present study, the novel SIRT2-specific inhibitors NCO-90/141 induced caspase-dependent cell death such as apoptosis or CICD such as autophagic cell death in leukemic cell lines. Although apoptosis and autophagy share many common mechanisms, current knowledge of the molecular interactions between autophagic and apoptotic pathways is incomplete and fragmented. Therefore, it may be necessary to further elucidate the relationship between apoptosis and autophagy following NCO-90/141 treatment in primary leukemic cells.

## Additional file

Additional file 1: **Figure S1.** AGK2 does not reduce cell viability of leukemic cell lines. Cell lines were incubated at 2 × 10^5^ cells/mL in the presence of various concentrations of AGK2 for 48 h. The viabilities of the cultured cells were measured by Cell Count Reagent SF. Cells cultured in the absence of AGK2 were assigned a relative viability of 1. Data represent mean percentages ± SD of three independent experiments. (TIF 532 kb)

## Abbreviations

AML: Acute myeloid leukemia
ATL: Adult T-cell leukemia/lymphoma
CICD: Caspase-independent cell death
HTLV: Human T-cell leukemia virus
TUNEL: Terminal deoxynucleotidyl transferase dUTP nick end labeling
